# A School-Based Intervention Using Health Mentors to Address Childhood Obesity by Strengthening School Wellness Policy

**DOI:** 10.5888/pcd16.190054

**Published:** 2019-11-21

**Authors:** Nisha Narayanan, Nikita Nagpal, Hillary Zieve, Aashay Vyas, Jonathan Tatum, Margarita Ramos, Team KiPOW!, Robert McCarter, Candice Taylor Lucas, Michele Mietus-Snyder

**Affiliations:** 1 Division of Pediatric Emergency Medicine, New York University Langone Medical Center, New York, New York; 2 Department of General Pediatrics, New York University School of Medicine, Bellview Hospital Center, New York, New York; 3 Department of Pediatrics, Children’s Hospital of Orange County, Irvine, California; 4 Department of General Pediatrics, Valley Children’s Healthcare, Irvine, California; 5 Department of General Pediatrics, Cincinnati Children’s Medical Center, Cincinnati, Ohio; 6 Department of General Pediatrics, Children’s National Hospital, Washington, District of Columbia; 7 Author members of Team KiPOW are listed in Acknowledgments; 8 Division of Biostatistics and Study Methodology, Children’s National Hospital, Washington, District of Columbia; 9 Department of Pediatrics, University of California, Irvine School of Medicine, Irvine, California; 10 Pediatric Exercise and Genomics Research Center (PERC), University of California, Irvine School of Medicine, Irvine, California; 11 Center for Translational Research, Children’s National Hospital, Washington, District of Columbia; 12 Division of Pediatric Cardiology, Children’s National Hospital, Washington, District of Columbia

## Abstract

**Purpose and Objectives:**

The objective of our study was to strengthen wellness policy in Title 1 schools by implementing a mentored behavior-change model that extends the continuum of care from academic to community settings and mobilizes existing public resources in accordance with US Preventive Services Task Force screening guidelines for childhood obesity management.

**Intervention Approach:**

Team Kid POWER! (KiPOW!) health mentors (students and trainees in medical and health-related fields) in 2 geographically and demographically distinct school districts, the District of Columbia and Orange County, California, delivered standardized health curricular modules to fifth grade classrooms, modeled healthy eating behaviors during school lunchtime, and engaged in active play at recess.

**Evaluation Methods:**

Initial interventions in the the District of Columbia and Orange County delivered 10 sessions in which all participants received the intervention. Two subsequent interventions in Orange County, for 5 weeks (Lite) and 10 weeks (Full), included controls. Pre–post measurements of body mass index (BMI) and blood pressure were documented in all participants. A mixed linear regression model, which included a random effect for each school, estimated differences between Full and Lite interventions compared with controls, adjusting for site, sex, and baseline status of the dependent variable.

**Results:**

KiPOW! Full, but not KiPOW! Lite, was associated with a modest reduction in BMI percentile compared with control (KiPOW! Full,* P* = .04; KiPOW! Lite,* P* = .41), especially in Orange County (*P* < .001). Systolic blood pressure improved in Full (*P* < .046) more than in Lite interventions (*P* = .11), and diastolic blood pressure improved in both Full (*P* = .02) and Lite (*P* = .03) interventions. Annual renewal of the school and volunteer commitment needed to maintain KiPOW! was found to be sustainable.

**Implications for Public Health:**

KiPOW! is a generalizable academic–community partnership promoting face-to-face contact between students and trusted health mentors to reinforce school wellness policies and foster youth confidence in decision-making about nutrition- and activity-related behaviors to achieve reduced BMI percentile and lowered blood pressure.

**Summary**

**What is already known about this topic?**

As the prevalence of pediatric obesity and its associated comorbidities climbs, novel evidence-based approaches are needed to achieve changes in behavioral health. The need is most urgent among socioeconomically disadvantaged youth, who are disproportionately affected by the obesity epidemic.

**What is added by this report?**

Team Kid POWER! (KiPOW!) is a school-based, mentored, behavior-change model developed in accordance with US Preventive Services Task Force guidelines to reinforce school health policy in 2 demographically and geographically diverse high-risk school environments. The program has shown favorable health outcomes.

**What are the implications for public health practice?**

This academic–community partnership promoting face-to-face exposure with trusted health mentors represents a feasible, replicable tool to combat obesity and its effects.

## Introduction

The prevalence of childhood obesity is increasing and disproportionately affects low-income and minority youth, who also face increased risk for associated cardiometabolic comorbidities (1). Social inequities are deeply entrenched in schools, the environment where children spend at least 5 days per week for most the year and where they consume approximately 35% to 50% of their daily calories (2). High-poverty school districts spend 15.6% less per student than low-poverty districts, an inequity recognized by the US Department of Education, partly because most school funding comes from local taxes (3).

Schools provide an important opportunity for lifestyle change that can either reinforce or transcend socioeconomic barriers to improved diet and physical activity (4). Recognizing this, the federal government supports 8% to 9% of school budgets. These funds are primarily dedicated to the National School Lunch Program, which, in accordance with the Healthy Hunger-Free Kids Act of 2010 (5), requires that free and reduced-price school meals be distributed preferentially to Title 1 schools (schools in which at least 40% of students are from low-income households). Nutrition standards in the program, which became effective in March 2012, align with evidence-based pediatric guidelines for preventing childhood obesity (Appendix) and are “expected to enhance the diet and health of school children, and help mitigate the childhood obesity trend” (5). School wellness policies support the federal standards locally, and in the the District of Columbia, the Healthy Schools Act of 2010 exceeds them (6).

Although proactive local and federal school policies to combat childhood obesity exist, implementation of them is incomplete in the District of Columbia (7) and elsewhere. Fruits, vegetables, and whole grains, the essential foods most often missing in the American diet across the lifespan, are the foods most often discarded in the cafeteria (8). Local school districts in the United States and Canada show wide discrepancies between physical activity and health education policy and performance (7,9). Therefore, although school wellness policies are evidence-based, they need a catalyst if they are to realize their ambitious goal of changing individual health behaviors. Although the conclusions of recent systematic reviews conflict as to whether school-based programs can (10) or cannot (11) stem the rising tide of childhood obesity, there is consensus that schools cannot do it alone (12), nor can the health care system, with its own set of competing priorities (13).

The US Preventive Services Task Force (USPSTF) guidelines on screening for childhood obesity management (14,15) recommend 26 contact hours of face-to-face time (hereinafter face time) with a trusted health care provider over a period of 2 to 12 months to achieve successful behavior change. This time is not readily attained in the traditional clinical setting. USPSTF 2010 recommendations were underscored in a 2017 update (16, with new emphasis on the need to “go beyond the clinician’s office” (17). Schools can be powerful community allies to health care systems by connecting health care providers directly with children to achieve the face time required to support healthy behaviors.

Alliance with health care advocates can also further the priorities of school stakeholders. The insulin resistance and cardiometabolic risk that accompany obesity and poor nutrition (18) are associated with cognitive dysfunction (19). A strong association between lifestyle behaviors linked to insulin dysregulation and student academic performance has been identified among Canadian fifth graders (20). Danish public school test scores between 2009–2010 and 2012–2013 showed that for every 20- to 30-minute break in the school day there was an incremental increase in average test performance (21). Thus, the investment of academic time toward improving the diet and activity behaviors highlighted in school wellness policies may also improve academic outcomes.

## Purpose and Objectives

Team Kid POWER! (KiPOW!) was developed in 2012 in the District of Columbia as an alliance among pediatric health care providers, medical students, and inner city school districts to advance common goals for student health and academic success. Our overarching purpose was to address the 5 key elements of the RE-AIM (reach, efficacy, adoption, implementation, and maintenance) framework (22) to evaluate KiPOW!’s effect as a real-world intervention designed to improve the implementation of federal and local school policies. We aimed to determine 1) KiPOW!’s feasibility and replicability in fifth-grade classrooms of geographically and demographically distinct Title 1 elementary schools in the the District of Columbia and Orange County, California, that were predominantly minority and 2) the intervention’s effect on improving body mass index (BMI) percentile and blood pressure.

## Intervention Approach

### Program design

Community engagement was fundamental to the development of KiPOW! at both locations. Local departments of education, school boards, participating school communities, school principals, and teachers were engaged through stakeholder meetings before each intervention year. Leadership from the host academic medical schools and children’s hospitals at both sites were informed about the program and included KiPOW! in federally mandated community benefit reports. The Safeway Grocery Foundation in the the District of Columbia and the local chapter of the American Academy of Pediatrics in Orange County were engaged through grant writing to help fund program costs.

Social cognitive theory (23) was used to create the conceptual framework of KiPOW!. This theory recognizes that human behavior is shaped by environmental, behavioral, and cognitive factors that can be influenced by observational learning, outcome expectations, and self-efficacy and the belief that one has the ability to adopt a behavior and have the desired outcome. Social cognitive theory aligns well with KiPOW!, which matches students with health mentors who demonstrate a social desirability for healthy behaviors and who serve as role models advocating for initiatives mitigating adverse social determinants of health.

**Figure 1:**
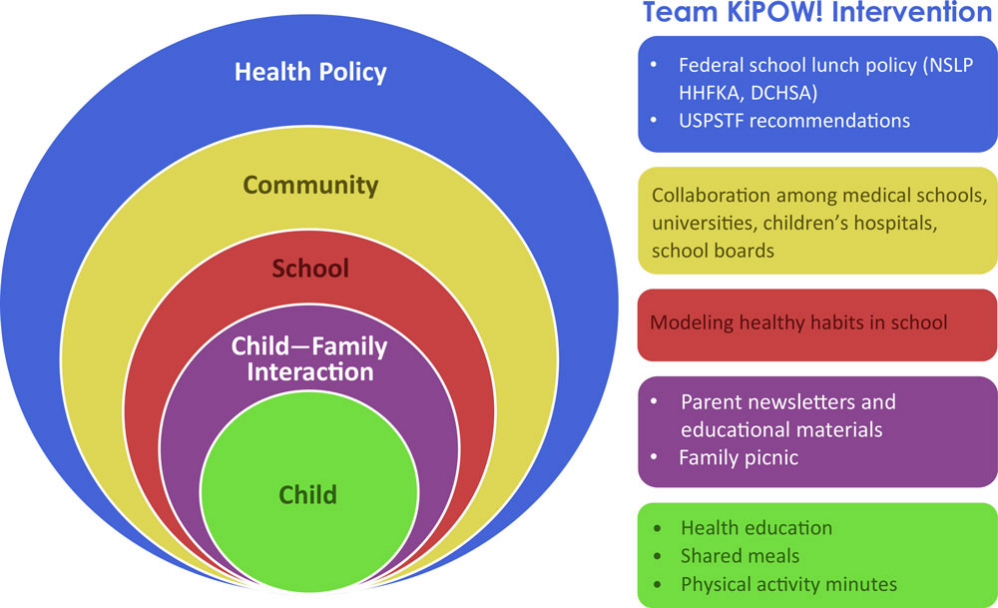
Team KiPOW! model. Components of the Team KiPOW! intervention and its multiple levels of interaction. KiPOW! is based on a socio-ecological model of health promotion and was developed with existing health policy in mind. Abbreviations: DCHSA, District of Columbia Healthy Schools Act 2010; HHFKA, Healthy Hunger-Free Kids Act; NSLP, National School Lunch Program; USPSTF, US Preventive Services Task Force.

KiPOW!’s program components were designed on the basis of the socio-ecological model of health promotion. The model considers the influence of the interrelationships between diverse social sectors and environmental factors on obesogenic behaviors (Figure 1). Recent US Department of Agriculture guidelines stated, “Everyone has a role in supporting healthy eating patterns” (24). KiPOW! capitalizes on this feature as a school-based academic–community partnernership for obesity prevention.

A quasi-experimental study design was used to assess KiPOW! in elementary schools in the District of Columbia and Orange County. In the District of Columbia, KiPOW! was conducted for 1 semester in 2 intervention schools with no control group. In Orange County, KiPOW! was conducted for 3 semesters in 3 schools: the first year in school 1 with no control class, the second year in school 1 with both an intervention and control class assigned by the school principal, and the third year in school 2 with a matched but distinct control class in school 3, assigned by the school district liason. Annual interventions continue at both sites with a new class of Team KiPOW volunteers each year. By design, control school 3 became the intervention school for KiPOW! Orange County following completion of its year as a control.

### Health mentors

In both the District of Columbia (George Washington University School of Medicine) and Orange County (University of California, Irvine, School of Medicine), medical student health mentors were recruited at orientation and by email, flyer, and word of mouth. In Orange County, premedical students and pediatric medicine residents were also recruited. All health mentors passed a background check before KiPOW! participation. Both groups were trained in evidence-based counseling in childhood obesity prevention (25), USPSTF childhood obesity screening recommendations (14–16), current school health policies, and the rationale for the mentored behavior-change model (24). This training was provided by the faculty pediatrician mentor in the District of Columbia and was replicated by pediatric medicine resident leaders in Orange County. Research design and methods were discussed. KiPOW! health mentors completed Human Subjects Collaborative Institutional Training Initiative modules on social and behavioral research and signed a contract agreeing to volunteer at least once a month in participating fifth-grade classrooms and to eat the same healthy school lunch as students, with a smile (26). Physical activity training addressed team building and conflict resolution. It included high-energy, large-group games and exercises designed to encourage maximum participation in active play by elementary school students during recess. In the District of Columbia, mentors received formal Playworks leadership training (27). In Orange County, a modified version of this training was provided. Both groups of mentors were given identical training manuals with a series of interactive games that were well received in the District of Columbia pilot year.

Fifty-six health mentors in the District of Columbia and 67 in Orange County participated in KiPOW! in the trials reported in this article. Mentors wore royal blue “superhero” techwear T-shirts with the Team KiPOW! logo prominently displayed on the front to every school session. Although visiting mentors varied by session, every mentor wore the KiPOW! T shirt, which engendered familiarity. Each site had 1 pre-med volunteer who attended every KiPOW! session.

## Intervention

**Figure 2:**
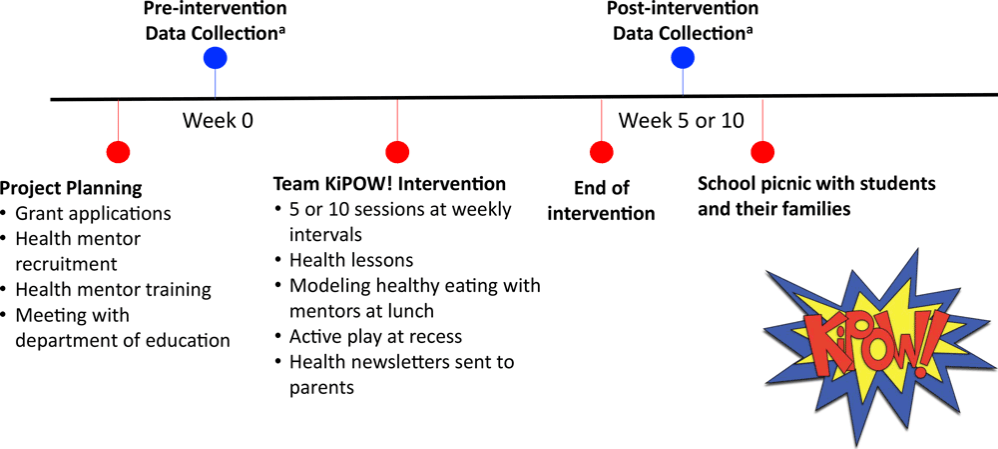
Team KiPOW! intervention timeline. Each Team KiPOW! intervention consisted of a project planning phase, pre-intervention data collection, the intervention itself, postintervention data collection, and a school picnic with students and their families. Data collection consisted of height, weight, blood pressure measurements, and possibly fitness assessment and behavior survey, depending on the session.

The intervention was standardized across all KiPOW! sessions at both sites (Figure 2). The medical student co-leader of the District of Columbia KiPOW! initiated and continued to co-lead KiPOW! Orange County during her pediatric medicine residency, ensuring standardization of the KiPOW! intervention. The only distinction was a shortened intervention from the usual 10 to 5 sessions in 1 semester in Orange County in response to a change in school administration priorities. We designated this shortened intervention KiPOW! Lite. Trained health mentors visited the intervention fifth grade students for 75 minutes per scheduled session on an approximately weekly schedule contingent on the host elementary school’s vacation and standardized test schedule.

At both sites, each KiPOW! session began with a mentor team meeting. Next, mentors conducted a short, evidence-based health lesson immediately before lunch. Lessons (Box) were pre-scripted, but mentors were instructed to encourage and incorporate classroom participation. Simple take-home messages were emphasized, and each lesson was accompanied by a colorful health newsletter sent home to parents, highlighting key points. Following the lesson, mentors accompanied their fifth grade class to the cafeteria and ate the school lunch with the children, modeling healthy lunch line choices. During recess, health mentors led children in active group games. Baseline data were collected before KiPOW sessions, and postintervention data were collected after.

**Box. Lesson Topics, Team KiPOW!, District of Columbia and Orange County, California, 2012–2017**

**Table:** 

Week	Lesson Topic (Take-Home Message)
1	Introduction to Team KiPOW!, My Plate Model (Smart Food + Active Play = ENERGY aka POWER)
2	Breakfast (One A Day)
3	Water (Introduction to the P-Meter – What Pee Color Tells You)
4	Exercise (Introduction to Blood Pressure – and How Food and Play Can Change It)
5	Fruits, Vegetables, and Vitamins (Eat the Rainbow)
6	Carbohydrates and Nutrition Labels (Why Fiber is a Carbohydrate Upgrade)
7	Proteins (Think Flexitarian) and Fats (Where to Find the Best Kind, Which to Leave Behind)
8	Snacks (How to Use What We Know Now to Feed a Snack Attack)
9	Sleep (Make Mindful Breathing Part of Your Night Time Routine)
10	Review (Jeopardy Game Show — Review)

Parents were invited to join KiPOW! at the end of the intervention for a picnic during which the children presented what they had learned, and shared with their parents how to make a healthy, balanced plate. Parent attendance was very good at both sites but not quantified.

## Evaluation Methods

From 2013 through 2017, KiPOW! was implemented in 5 schools (2 in the District of Columbia and 3 in Orange County), delivered a shared health promotion curriculum in 4 (1 control school excluded from curriculum), and measured common outcomes in all. No a priori sample size calculations were made for this exploratory community collaboration. Inclusion criteria at both sites were 1) Title 1 status elementary school, and 2) permission from the schools for KiPOW! health mentors to teach a 25-minute curricular module before lunch, then to stay with fifth graders through the lunch and recess periods. Human subjects approval was obtained from institutional review boards of the Children’s National Hospital (the District of Columbia) and the University of California, Irvine. Parallel local school board research approval was also obtained, from the Office of the State Superintendent of Education in the District of Columbia and the Orange County Board of Education in Orange County.

Quantitative evaluation methods were used. In the District of Columbia, because outcome assessments were limited to measures already obtained in the schools, consent and assent waivers were granted. Participating students and their parents were given an information sheet about the program. In Orange County, because an adaptation of the HABITS behavioral questionnaire was introduced (28), a parental opt-out consent was provided. All children in the school’s pre-designated intervention classes in both the District of Columbia and Orange County participated in the KiPOW! intervention. Aware of the impact that KiPOW! had on their own career trajectories, self-assessment of the team KiPOW! experience was initiated by study authors by using Donald Kirkpatrick's four-level training evaluation model (reaction, learning, behavior, results) (29). Institutional review board approval was obtained to send an anonymous email survey generated with Research Electronic Data Capture (REDCap, Vanderbilt University) to current and past KiPOW! health mentor volunteers in the District of Columbia and Orange County about their experience with the program and its effect on their current clinical interactions and subsequent careers.

## Outcome Measures

Baseline and post-intervention quantitative measures (weight, height, and blood pressure) were obtained, with attention to privacy and confidentiality, from all fifth-grade participants in intervention and control schools in the same school semester of the KiPOW intervention. In control schools, however, visiting KiPOW! volunteers did not wear KiPOW! T-shirts while obtaining measures.

A written measurement protocol was followed at both sites. In the District of Columbia, KiPOW! medical students assisted school nursing personnel in taking pre–post measures of weight, height, and blood pressure. A mechanical column scale and stably installed stadiometer were available in a private corner of the school nursing suite in each District of Columbia school. In Orange County, KiPOW! health mentors measured height by using a measuring tape attached to the wall, and weight measures were obtained by using a calibrated floor scale temporarily installed in a private space. At both sites, mentors measured blood pressure in the right arm by using Omron portable monitors with either a small (child-sized) or regular (adult-sized) cuff, as appropriate, so that the cuff bladder covered 75% to 80% of the student’s upper arm circumference. The cuff size used was recorded at the baseline visit, and the same cuff size was used at follow-up. Each subject rested seated for 5 minutes before obtaining 2 blood pressure readings. A third measure was taken if either the systolic blood pressure (SBP) or diastolic blood pressure (DBP) reading of the first 2 differed by more than 5 mm Hg. An average of the 2 measures closest to each other was taken.

In all Orange County interventions, health behavior variables were queried by using the 19-item HABITS questionnaire previously validated for participants aged 7 to 16 (28) with 2 additional questions about physical activity (participation in organized sport and number of days and average hours on those days active outside of school). The modified HABITS questionnaires (28) were completed by the fifth graders for both the intervention and control groups on the same days that pre–post study measures were obtained. Questionnaires were returned anonymously to a common pile, face-down.

### Statistics

We entered de-identified baseline and study completion data points, paired by student participant study identification, into Stata version 13.1 (StataCorp LLC). We reported descriptive statistics as means and standard deviations (SDs) for students grouped by their participating school. We tested differences between groups on categorical baseline variables by using generalized estimating equations with school as a cluster variable, and by controlling for baseline status.

The intervention effect was analyzed for students with both pre and post data available. The main outcome variables were BMI percentile and SBP and DBP, because these were available at baseline and study completion for all study sessions. A mixed multiple linear regression model included a random effect for school. The model also took into account whether the participant attended an intervention or control school and whether the student was in the District of Columbia or Orange County. Site served as a surrogate for race and ethnicity in the study schools. The model controled for the before status on each outcome and for participant sex. Both Full and Lite intervention effects were compared with consideration of whether there was a dose response.

## Results

The KiPOW! model was implemented successfully with outcomes assessment in 2 distinct underserved populations over 4 academic years. It was warmly received by elementary school students, staff members, and administration in both locations. Health mentor engagement was sustainable from year to year. Past and current KiPOW! volunteers (N = 84) responded to an anonymous online survey about their experience with the program and its effect on their current clinical interactions and subsequent careers. Most volunteers reported positive feedback from school teachers (91%), from school administration (93%), and from fifth-grade mentees (99%). The balance of replies to these 3 queries (9%, teachers; 7%, administrators; and 1%, mentees) were neutral. Ninety-five percent of respondants agreed that they were likely to counsel their patients about lifestyle behaviors, of whom 68% reported that they were more likely to do so than before KiPOW!; 32% reported no change.

**Figure 3:**
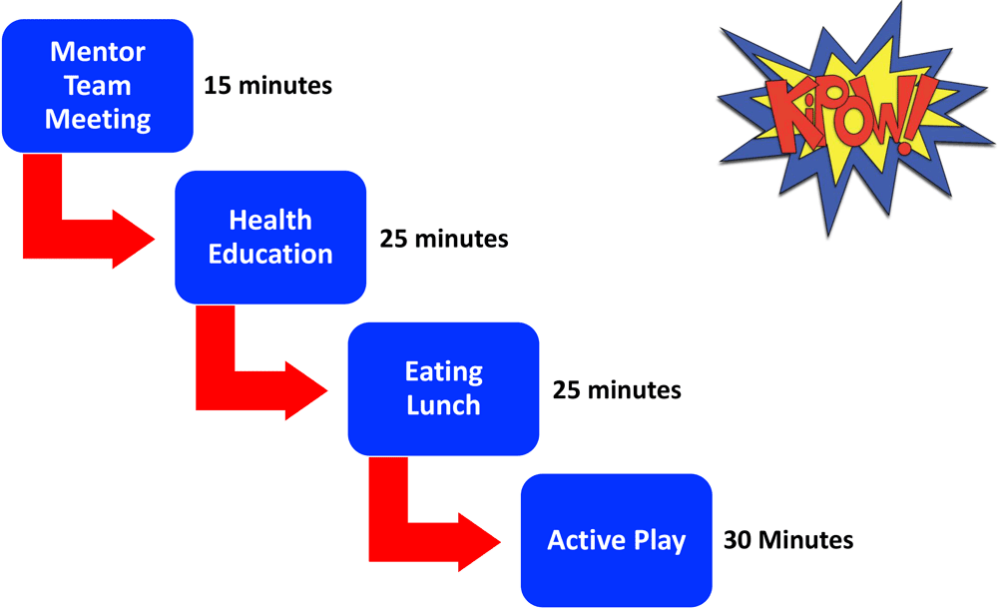
Team KiPOW! session schedule. Each Team KiPOW! weekly intervention session consisted of these 4 components; however, the order of eating lunch and active play differed per location.

The face time with Team KiPOW mentors delivered with each session (Figure 3) provided participating school children with an additional 6.25 hours of healthy lifestyle modeling over the 5-week and 13.3 hours over the 10-week trials, reinforcing local and national health policies aligned with American Academy of Pediatrics–recommended diet and exercise goals.

**District of Columbia**. KiPOW! was piloted in the District of Columbia in 10 sessions from January through May 2013. In 2013, fifth graders (n = 57) were enrolled from classes in 2 different urban Title 1 schools (Table 1). Almost all (97%–99%) District of Columbia participants were non-Hispanic black, and 100% of the school population qualified for free and reduced-price lunch. KiPOW! has been implemented annually in the District of Columbia since this introductory pilot, but outcomes assessments have not been obtained.

**Table 1:** Baseline Characteristics, Fifth Graders in Schools Participating in Team KiPOW! in the District of Columbia and Orange County, California, by Year, 2012–2017

Site	District of Columbia		Orange County				
Year	2013	2013	2015	2016	2016	2017	2017

School	School 1	School 2	School 1	School 1-I	School 1-C	School 2-I	School 3-C
Sessions, no.	10	10	10	5	5	10	10
Participants,^a^ no.	34	23	54	51	35	84	80
Age, y, mean (SD)	10.9 (0.6)	10.9 (0.4)	10.7 (0.4)	10.1 (0.3)	10.1 (0.2)	10.2 (0.4)	10.3 (0.5)
Female, %	52.9	34.8	46.3	51.8	59.5	51.8	47.5
BMI percentile, mean (SD)	74 (28)	71 (26)	74 (25)	80 (26)	86 (19)	67 (31)	79 (25)
BMI >97th percentile, %	29	13	11	23	34	14	21
Systolic blood pressure, mean (SD)	102 (7)	110 (11)	111 (13)	111 (16)	91 (10)^b^	107 (12)	111 (12)
Diastolic blood pressure, mean (SD)	65 (6)	67 (9)	71 (7)	71 (11)	70 (9)	68 (9)	69 (9)

Abbreviations: BMI, body mass index; SD, standard deviation.

a Although demographic characteristics of students in schools at each site were similar, the 2 sites differed overall. In the District of Columbia, 97% to 99% of students were non-Hispanic black, and 100% qualified for free and reduced-price lunch. In Orange County, 89% to 94% were Hispanic, and 67% to 86% qualified for free and reduced-price lunch.

b The only significant baseline difference between the Orange County school 1 intervention and control classes in 2016 was in systolic blood pressure.

**Orange County**. Three consecutive KiPOW! interventions were implemented from January 2015 through June 2017 in 2 Orange County Title 1 schools where 89% to 94% of children were Hispanic and 67% to 86% of the school population qualified for free and reduced-price lunch. In 2015, in the first Orange County School, 54 fifth graders participated, and all received the intervention. In 2016, the same school participated but the intervention was delivered to only 1 class of 51 while another class of 35 students served as controls (KiPOW! Lite), and in 2017, 2 separate schools joined. The intervention school had 84 participating 5th graders, and the control school had 80 participating students. Intervention and control schools in 2017 were in close geographic proximity and had similar demographics (Table 1).

Eight students were absent on data collection days and therefore not available for follow-up, 4 in the District of Columbia and 4 in Orange County. All 8 had similar baseline characteristics as retained participants; 50% were female.

### Baseline health metrics

We found no significant difference at baseline in age, sex, and BMI percentile between schools in the District of Columbia and Orange County, nor among Orange County intervention and control schools (Table 1). The mean BMI percentile was skewed right in all classes of participating fifth graders. Similarly, mean SBP and DBP were slightly above the 50th percentile (30) for age, height, and sex in all children, with the exception of a significantly lower baseline SBP in the control class for Orange County school 1 in 2015–2016.

### Observed changes with the KiPOW! intervention

The KiPOW! intervention predicted a modest reduction in BMI percentile of 0.05 units for the Full intervention in comparison with controls (*P* = .04) (Table 2). The difference between Lite and Control interventions did not meet significance. We saw a difference by site in BMI percentile change, with greater reduction in Orange Count than in the District of Columbia (*P* < .001), but a very low intraclass correlation (ICC = 0.001) of the effect by school.

**Table 2:** Comparison of Changes in Body Mass Index Among Fifth Graders in Schools Participating in Team KiPOW! in the District of Columbia and Orange County, California, 2012–2017^a^

Factor	Intervention		*P *Value^b^
	Coefficient	Post Group, Mean (SE) [CI]	
**Body mass index, percentile, %**			
Full^c^	−0.05	71.7 (1.14) [69.5–74.0]	.04
Lite^d^	−0.03	73.7 (2.01) [69.7–77.6]	.41
Control		75.7 (1.48) [72.8–78.6]	Reference^e^
**Systolic blood pressure**			
Full^c^	−7.2	107 (2.2) [103–111]	.046
Lite^d^	−7.2	107 (3.7) [100–114]	.11
Control		113 (2.7) [109–119]	Reference^e^
**Diastolic blood pressure**			
Full^c^	−3.0	67 (0.7) [65.3–67.9]	.02
Lite^d^	−3.3	66 (1.2) [63.9–68.6]	.03
Control		70 (0.9) [67.7–71.3]	Reference^e^

Abbreviations: CI, confidence interval; SE, standard error.

a Orange County and District of Columbia data were pooled in this regression model.

b Multiple regression model exploring predictive variables of dependent variables, adjusted for the baseline status of the variable, together with sex and geographic region (a surrogate for race/ethnicity), and comparing the Full KiPOW! 10-week intervention or the Lite 5-week intervention to the control group, which had no KiPOW! intervention.

c KiPOW! Full session with 10 weeks of intervention.

d KiPOW! Lite session with 5 weeks of intervention.

e Control group was used as the reference in this regression model.

Both SBP and DBP readings also improved in Full intervention schools compared with control schools (SPB, *P* = .046 ; DBP, *P* = .02. Only DBP readings improved significantly following the Lite intervention (*P* = .03); SBP values fell following the Lite intervention but did not reach significance (Table 2). SBP fell an average 7.2 units in both Full and Lite interventions. DBP dropped an average of 3.0 units in the Full and 3.3 units in the Lite internvention. The reduction in SBP was greater in the District of Columbia than in Orange County with a modest ICC by school of 0.09. There was no difference by site in DBP change, and ICC was low (ICC = 0.03).

A more comprehensive behavioral assessment was undertaken in the 3 Orange County study cohorts by using the modified HABITS questionnaire. In the Orange County year 1 intervention, increased water and decreased sweetened beverage intake were reported, but these results were not replicated in subsequent years. Minutes reported in active exercise trended up in Orange County school 1 during the Full year 1 and Lite year 2 interventions, but not in controls.

## Implications for Public Health

The real-world implementation potential of this program is illustrated through the RE-AIM framework (22). The reach of KiPOW! extends to Title 1 elementary schools whose students are socioeconomically disadvantaged and at heightened risk for obesity and who stand to benefit most from improved access to healthy lifestyle behaviors. Efficacy is demonstrated by improvement in BMI percentile and blood pressure outcomes attainable in the school setting. Consistent with social cognitive theory and the socio-ecological model, students and trainees in medical and health-related fields can serve as trusted health-behavior mentors to elementary school students to help them achieve these outcomes. The replication of KiPOW! in 2 geographically and demographically distinct school districts indicates its potential for the RE-AIM adoption component in other Title 1 schools in proximity to medical and other health-related training programs. More than half (53%) of respondents to the KiPOW! mentor survey expressed interest in creating a KiPOW! chapter in the next city where they practice medicine. This response suggests that it will be possible going forward to evaluate the external validity of KiPOW! in multiple school settings. KiPOW! was designed to leverage the strengths that each partner can sustainably provide to achieve common goals, and our early experience indicated that the program has been viewed positively by mentors, education stakeholders, and school children alike. Cost-effective implementation of existing school wellness policy with KiPOW!’s mentored behavior-change model contributes face time toward the evidence-based USPSTF recommendations for screening and management of childhood obesity. The same guidelines and standards were used to achieve this outcome in 2 environments. Finally, the mobilization of renewable and sustainable volunteer health professional student energy demonstrates that this program can be sustainably maintained.

Behavior change is as chronic a challenge as the many diseases engendered by failure to change behavior. Fewer than 1% of American children and adolescents meet recommended dietary guidelines (31,32). Three-quarters of American youth fail to meet the recommended 60 minutes of daily physical activity (33), which adds to the cardiometabolic risk created by poor nutrition (34). An estimated 45% of all cardiometabolic deaths in the United States (deaths from heart disease, stroke, and diabetes) are associated with suboptimal diets (35), underscoring the potential for progress in this domain. Although modest improvements have occurred in the American diet over the past decade, notably consumption of less trans fat and fewer simple carbohydrates (less sugar-sweetened beverage intake, more whole grains) these improvements are concentrated among people of middle and high socioeconomic status (36), which only widens the gap for people already at heightened risk (36). As the prevalence of childhood obesity climbs, our overburdened healthcare system needs to find novel ways of achieving evidence-based behavior change and health equity. Because school wellness policy aims to improve the child nutrition and activity behaviors targeted in childhood obesity prevention and management guidelines (15,16,25), common ground exists for collaboration between the health and education sectors.

KiPOW! demonstrates that a school-based, mentored, behavior-change model that is in accordance with USPSTF guidelines and designed to strengthen the implementation of public school wellness policy can advance child health outcomes.

Despite consensus that schools represent an ideal access point for health behavior interventions to combat childhood obesity (38), results have been mixed. Systematic reviews of multiple small and varied school-based interventions have suggested short-term benefits for obesity prevention (10,39); however, 2 large randomized, prospective, multisite elementary school trials in United States (HEALTHY) (40) and Europe (WAVES) (12) failed to achieve significant overall change in BMI percentile. The HEALTHY study did, however, report improved overall indexes of adiposity and improved BMI *z*-scores among the subset of youth at highest baseline levels.

A recent review suggested an implementation strategy focused on evidence-based behavior changes rather than BMI might help avoid the bias and stigmatization that have been associated with school-based obesity prevention interventions (41). KiPOW! delivers an evidence-based curriculum that is weight neutral, focused instead on the energy and learning power unleashed with healthy lifestyle choices. Other barriers to the sustainability of school-based interventions include cost and time limitations (41). Because of its reliance on volunteer health mentors, implementation cost is low. One of KiPOW!’s greatest strengths may be its sustainable volunteer energy, renewed annually with incoming students in health-related fields and medical school who are motivated to provide the recommended USPSTF face time with elementary students that is so difficult to achieve in the traditional clinical setting. The good will engendered from schools and school children and reported by the volunteer KiPOW! mentors reinforces this energy. In addition, health mentors who volunteer for KiPOW! learn about both childhood health promotion and the challenges of behavior change. These reciprocal effects of participating in KiPOW! offer a valuable supplement to traditional medical education. The program’s overarching aim —strengthening the implementation of proactive and fully funded school policy — aligns the intervention with school priorities. By focusing on outcomes readily available in participating schools and by working largely in the school lunch and recess periods, KiPOW! minimizes its demand on one of the most limited school site resources — academic time. The program is ongoing, supported at both sites by an academic medical center and a strong board of volunteers with distinct organizational roles and transition protocols. The replicability of KiPOW! in 2 different school districts suggests that it may be possible to continue to expand and sustain the program.

Our pilot study of KiPOW! had several limitations. It involved only fifth-grade classrooms in 2 schools in the District of Columbia and 3 schools in Orange County. The interventions were limited to 5 to 10 weeks. In addition, by design, all schools were Title 1 elementary schools, and our findings may not apply to higher-resource schools and to other grades. Despite involving parents with weekly newsletters and a family participatory meal at the end of the semester, KiPOW! was limited in its ability to involve families and the wider school community to help sustain the benefits accrued. School absences during the KiPOW! intervention were not measured and therefore not accounted for in the results. As is typical in school-based research, variations in local school resources necessitated slight site-specific modifications in the study design, although fidelity to the program model was maintained. Our main findings were limited to primary outcomes, which are readily available in the school setting, to minimize academic disruption. Self-report behavioral data in the HABITS questionnaires used in Orange County may have been affected by social desirability bias. The challenge of obtaining self-report behavioral data from fifth-grade participants underscored the need for more objective lifestyle assessment tools. Thus, the factors influenced by KiPOW! most likely to mediate the observed favorable health outcomes remain to be defined.

Although the ability to document improvement in behavior choices was limited, successful community engagement and favorable health outcomes in 2 geographically and demographically distinct school districts are encouraging and support further systematic evaluation of this promising academic–community collaborative. Ongoing KiPOW! interventions are planned to examine the effect of more frequent visits that can meet or exceed the USPSTF minimum recommendation of 26 hours of face time with a health care provider. Ongoing research is evaluating the effect of KiPOW! on school teachers, administrators, parents, and school children in existing and additional sites. Further research is needed to determine how to more effectively engage teachers, parents, and the community with innovative methods, such as digital outreach.

In summary, a school-based intervention promoting face-to-face mentoring may be a feasible adjunct to reinforce school wellness policy in inner-city school districts near colleges and universities with medical schools and other health-related programs. Efforts to standardize implementation methods via a national KiPOW! toolkit, improve data collection and outcomes assessments, and replicate KiPOW! in additional school districts across the United States and Canada are currently underway.

## Appendix

This appendix is available for download as a Microsoft Word file.
