# Supplementary material for: A School-Based Intervention Using Health Mentors to Address Childhood Obesity by Strengthening School Wellness Policy
Source: Prev Chronic Dis. 2019 Nov 21;16:E154. doi: 10.5888/pcd16.190054 (PMC6880918; doi:10.5888/pcd16.190054)
Supplement: Supplementary file 1 [file 19_0054_appendix.docx]

Appendix

Comparison of School Policy Tenets from the American Academy of Pediatrics, the District of Columbia Healthy Schools Act, and the National School Lunch Program Healthy, Hunger-Free Kids ACT

| **Category** | **American Academy of Pediatrics Expert Recommendations Regarding the Prevention, Assessment, and Treatment of Child and Adolescent Overweight and Obesity (2007)(25)^a^** | **C Healthy Schools Act (2010)(43)** | **National School Lunch Program Healthy, Hunger-Free Kids Act of 2010, (5) California State Board of Education Policy #99–03, Physical Education Standards for California Public Schools (2005)** |
| --- | --- | --- | --- |
| Diet | - Limit consumption of sugar-sweetened beverages - Encourage >5 servings of fruits and vegetables/ day - Limit eating out, particularly fast foods - Encourage family meals in which parents and children eat together - Limit portion size- visualized with the new USDA MyPlate model - Eat a diet high in fiber and rich in calcium and balanced macronutrients - Restrict consumption of highly processed, calorie-dense, nutrient-poor foods - Eat breakfast daily | 1. Include more whole grains, more variety of fruits and vegetables, and less fat, juice and sodium in school meals:  - Only 100% juice counts as a fruit serving only once per week - Fat-free or low-fat milk only must be offered every day - Vegetables must be offered every day with a different vegetable every day of the week - Fruits must be offered every day with a different fruit every day of the week - Fresh fruit must be served at least 2 days per week - Whole grains must be served at least once each day - Breakfast and lunch must contain limited saturated fat (<10% of calories), no trans fat, and by the year 2020 sodium will be restricted in bulk purchased foods - Schools are encouraged to serve a vegetarian option each week  1. Universal breakfast in Title 1 DC Schools 2. Promote healthy eating to students, staff, faculty, and parents; and solicit input from the parties above to design nutritious and appealing meals 3. Post menus, ingredients, and food origins in school offices and on websites 4. Encourage schools to serve locally grown fruits and vegetables 5. Improve nutrition of competitive foods (available in vending machines, snack bars) | - Offer fluid milk that is fat-free (unflavored and flavored) and low-fat (unflavored only) - Offer fruits and vegetables as 2 separate meal components - Offer fruit daily at breakfast and lunch (juice counts as a fruit) - Offer vegetables daily at lunch, including specific subgroups weekly (dark green, orange, legumes, and other) and a limited quantity of starchy vegetables - Offer whole grains: half of grains whole grain–rich upon implementation of the rule and all grains whole grain–rich within 2 years - Offer a daily meat/ meat alternate at breakfast - Adjust meal calorie ranges for each age/grade group - Reduce the sodium content gradually over a 10-year period through 2 intermediate sodium targets at 2 and 4 years - Prepare meals using zero grams of trans fat per serving - Require students to select a fruit or a vegetable as part of the reimbursable meal - Use a single food-based menu planning approach - Use narrower age/grade groups for menu planning - Require state agencies to:  1. Conduct a nutritional review of school lunches and breakfasts as part of the administrative review process 2. Determine compliance with the meal patterns and dietary specifications based on a review of menu and 2-week period 3. Review school lunches and breakfasts every 3 years, consistent with the HHFKA |
| Physical Activity | - Limit television and other screen time (≤2 hours/day; none for <2 years old)a - Encourage 1 hour of moderate to vigorous activity daily | 1. Grades K-5:   - - At least 30 minutes/ week for school years 2010–2011 to 2013–2014   - 150 minutes/ week for school year 2014–2015 and beyond   Grades 6–8:   - - At least 45 minutes/week for school years 2010- 2011 to 2013–2014   - 222 minutes/week (school year 2014–2015 and beyond)   2. Devote at least 50% of physical education class time to physical activity  3. Physical activity should be moderate to vigorous as much as possible  4. Provide suitably adapted physical education for students with disabilities  5. Not require or withhold physical activity as a punishment for students  6. Direct the Department of Parks and Recreation to provide schools with access to recreation centers, fields, playgrounds, and other facilities  7. Schools can encourage students to walk or bike to school, promote active recess, include physical activity in afterschool and classroom activities, and support athletic programs | - 200 minutes of physical education every ten school days for students in grades 1–6 (20 minutes/day) - 400 minutes of physical education every ten school days for students in grades 7–12 (40 minutes/day) |
| Health Education | - Not addressed | - Provide at least 15 minutes of health education per week to students in grades K–8 - By the 2014–2015 school year, schools must provide 75 min of health education per week to grades K–8 - Health education curricula and programs should include topics such as nutrition, mental health, sexual health, and drug abuse, and must continue to meet DC educational standards for health education - Ensure that students have adequate personal health knowledge (must be able to meet state health education standards by the end of the year) | - Not addressed |

^a^ Guidelines in place during the Team KiPOW! intervention years reviewed here have since been supplanted with the American Academy of Pediatricians Media Guide (42).
